# Burnout in Medical Residents: A Study Based on the Job Demands-Resources Model

**DOI:** 10.1155/2014/673279

**Published:** 2014-10-30

**Authors:** Panagiotis Zis, Fotios Anagnostopoulos, Panagiota Sykioti

**Affiliations:** ^1^Department of Neurology, Evangelismos General Hospital, 10676 Athens, Greece; ^2^Department of Psychology, Panteion University of Social and Political Sciences, 17671 Athens, Greece; ^3^South London and Maudsley NHS Trust, London SE5 8AZ, UK

## Abstract

*Purpose*. Burnout is a prolonged response to chronic emotional and interpersonal stressors on the job. The purpose of our cross-sectional study was to estimate the burnout rates among medical residents in the largest Greek hospital in 2012 and identify factors associated with it, based on the job demands-resources model (JD-R). *Method*. Job demands were examined via a 17-item questionnaire assessing 4 characteristics (emotional demands, intellectual demands, workload, and home-work demands' interface) and job resources were measured via a 14-item questionnaire assessing 4 characteristics (autonomy, opportunities for professional development, support from colleagues, and supervisor's support). The Maslach Burnout Inventory (MBI) was used to measure burnout. *Results*. Of the 290 eligible residents, 90.7% responded. In total 14.4% of the residents were found to experience burnout. Multiple logistic regression analysis revealed that each increased point in the JD-R questionnaire score regarding home-work interface was associated with an increase in the odds of burnout by 25.5%. Conversely, each increased point for autonomy, opportunities in professional development, and each extra resident per specialist were associated with a decrease in the odds of burnout by 37.1%, 39.4%, and 59.0%, respectively. *Conclusions*. Burnout among medical residents is associated with home-work interface, autonomy, professional development, and resident to specialist ratio.

## 1. Introduction

Over the last years, awareness that resident doctors are vulnerable to burnout has grown [1]. Residency is a stressful period in a physician's development where skills in a specialty need to be gained while simultaneously quality patient care is expected to be provided, leading to high levels of responsibility coupled with low levels of autonomy. Resident burnout may be a major problem; for example, burnout prevalence rates among US internal medicine residents have been found to range between 47% and 61% near the end of internship [2] while 58% of residents are burned out during their third year [3]. Rates for surgery residents are also very high, ranging from 47% to 70% [4]. Consequences of resident burnout may include self-reported suboptimal patient care, increased perceived medical errors, and deferred clinical decision making [5–7].

Burnout is a prolonged response to chronic emotional and interpersonal stressors on the job and is defined by the three dimensions of emotional exhaustion, depersonalization (cynicism), and reduced personal accomplishment (feelings of inefficacy) [8]. Emotional exhaustion (EE) refers to feelings of being overextended and depleted of one's emotional resources. Depersonalisation (DP) is characterised by a negative, cynical, and detached response to other people, including colleagues and patients or clients. A reduction in personal accomplishment (PA) occurs when a person feels less competent in his or her work [9].

Some debate exists regarding how to define burnout. Using on Maslach's Burnout Inventory (MBI), some investigators have defined burnout as high scores on EE and DP, combined with low scores on PA [9–11]. However, others argue that a better definition would be high scores on both EE and DP subscales [2] given that the PA subscale may measure a quality distinct from the other two [3]. Others still argue that the best way to define burnout is by considering high scores on either the EE or the DP subscale [3, 12].

However, while MBI has demonstrated its usefulness when it comes to measuring the dimensions of burnout, it does not provide information about areas in work-life that may contribute to a possible burnout. For this reason, Leiter and Maslach [13] created the questionnaire “Areas of Work-life Scale” (AWS) which measures both the opposing dimensions of burnout—Energy, Implication, and Efficacy—and the areas of work that could contribute positively or negatively to these three dimensions. This model of organizational context of burnout [14] is very consistent to the job demands-resources (JD-R) model, proposed by Demerouti et al. in 2001 [15], assumes that burnout can develop when job demands are high and job resources are limited, as such negative working conditions can lead to energy depletion and undermine employees' motivational potential, respectively. Job demands refer to those physical, social, psychological, or organizational aspects of the job that require high efforts from which the employee may be unable to recover adequately. Examples include work overload, time pressure, emotional demands, and work-home interface. Job resources refer to those aspects of the job that are instrumental to achieve work goals, may reduce job demands, or promote personal growth, learning, and development. Examples include autonomy, supportive colleagues and supervisor, performance feedback, and opportunities to learn [15].

Although burnout research has its roots in the care-giving and service occupations [8], burnout can be present irrespective of the type of profession. This phenomenon may be of special importance in health-care providers [16] because of the fact that burnout can lead to medical negligence and malpractice litigation, as well as suboptimal patient care practices and attitudes [17]. Previous studies have documented numerous correlates of resident burnout including excessive work hours, work overload, work-home conflict, and low support at work [18]. However, the studies that describe burnout syndrome and related factors solely among medical residents are limited, as most research has been carried out among doctors, irrespective of level of experience. Moreover, the samples of the studies are small or represent a single specialty, making it difficult to identify differences between specialties [1].

The purpose of our cross-sectional study was to analyze the burnout rates among medical residents in the largest Greek hospital and identify determinants based on JD-R model, using multivariate statistics. It was hypothesized that job burnout would be highest when job demands were high and job resources were low.

## 2. Methods

### 2.1. Procedure and Participants

“Evangelismos,” the largest Greek hospital in the National Healthcare Service, located in the capital city of Athens, with a capacity of 960 beds, covering 25 different specialties and affiliated with the University of Athens Medical School, was selected for this study. Eligible to take part in the study were all full-time residents who were appointed at least three months prior to the survey period and who were participating in the on-call rota.

An anonymous questionnaire was administered to all eligible candidates who were asked to return the completed questionnaires in a sealed envelope that was placed in a nontransparent empty box by the participant, in order to ensure the anonymity of the questionnaire. The principal investigator visited again the candidates one week after the initial distribution of the questionnaire to remind them about it and a second reminder visit took place one month later by the same person.

Participation in the study was voluntary. There was no interference in the study by anyone outside the team of researchers, which means that no pressure was applied by clinical or educational supervisors, managers or others to either take part in or ignore the study.

### 2.2. Survey Measures

#### 2.2.1. Sociodemographics

Sociodemographic details included age, gender, and marital status.

#### 2.2.2. Job Demands and Resources

Job demands were assessed via a questionnaire that included 17 items, which were rated on a Likert scale (ranging from “never” to “always”) according to the frequency with which the respondent felt a particular way in relation to his or her work. These 17 items were grouped into four factors: emotional demands (6 items, e.g., “Is your job emotionally hard?”), intellectual demands (3 items, e.g., “Do you consider your job as intellectually overwhelming?”), workload (4 items, e.g., “Do you work under time pressure?”), and home-work demands' interface (4 items, e.g., “Do you have to cancel personal appointments because of your professional commitments?”). The items for home-work demands' interface were based on those of Geurts et al. [19] and the items for the other three factors were based on those used by Bakker et al. [20]. The survey items were subsets of already validated instruments and constituted complete subdomains that were part of the original instruments, extracted en bloc.

Job resources were assessed via a questionnaire that included 14 items, which were rated on a Likert scale according to the frequency with which the respondent felt a particular way in relation to his or her work. These 14 items were grouped into four factors: autonomy in the job (3 items, e.g., “Can you decide on your own, how to prioritize your schedule”?), opportunities for professional development (3 items, e.g., “Do you feel that your job gives you the chance to learn new things?”), support from colleagues (3 items, e.g., “Can you ask your colleagues for help, when necessary?”), and supervisor's support (5 items, e.g., “My supervisor gives me feedback”). All items were based on those used by Bakker et al. [20].

The scales were translated from English into Greek, using a forward-backward technique. The Greek version of the instrument was then given to a small group of health professionals, and interviews were conducted with them, in order to ensure the clarity and comprehensibility of the items. No further changes took place.

#### 2.2.3. Other Work-Related Characteristics

Other work-related characteristics included type of specialty, years between graduation and beginning of specialty training, true working hours per day, number of on-calls per month, number of residents in department, number of specialists in department, and number of patients in department.

From the above mentioned information, the mean working hours per week could be calculated, allowing us to determine whether the European working time directive (EWTD) was violated or not. The directive lays down provisions for a maximum 56-hour working week (including overtime), a rest break after six consecutive hours' work, rest periods of at least 11 hours consecutive hours per 24-hour period, and a minimum of four weeks' paid leave per year, to protect workers from adverse health and safety risks. Moreover, we could easily calculate the residents per specialist ratio and the patients per resident ratio. Finally, in order to use it in the multivariate analysis we grouped the 25 different specialties into three large groups: medical, surgical, and other (including diagnostic and laboratory specialties) specialty groups.

#### 2.2.4. Burnout

The Maslach Burnout Inventory (MBI) was used to measure burnout. MBI is a validated 22-item questionnaire that is considered to be a standard tool for measuring burnout [8] and has been translated and validated in Greek [21].

Items on the MBI are rated on a 7-point Likert scale ranging from 0 to 6 according to the frequency with which the respondent feels a particular way in relation to his or her work (0 = never, 1 = a few times a year or less, 2 = once a month or less, 3 = a few times a month, 4 = once a week, 5 = a few times a week, and 6 = every day). The MBI yields three subscale scores that assess burnout in relation to its three dimensions: EE, DP, and PA.

Classification into low and high scores has usually been based on established cutoff scores [11, 21]. In their review on burnout in medical residents, Prins et al. [18] suggested that “the most effective way of diagnosing burnout seems to involve using a system of high scores on both EE and DP, or a high score on EE combined with a low score on PA. Scores ≥ 75% are considered high and scores ≤ 25% are considered low.” In keeping with these and others' recommendations [22], we defined burnout as a high score on EE, accompanied by high DP or low PA (i.e., “EE + 1” criterion). Given that there were no established burnout cutoff scores for the Greek version of the MBI, each distribution of scale scores was divided into quartiles, and high scores meant scoring in the 75th percentile or higher, whereas low scores meant scoring in the 25th percentile or lower. Thus, a high score on both MBI-EE and MBI-DP, or a high score on MBI-EE combined with a low score on MBI-PA were used to distinguish “burned out” from “nonburned out” respondents.

### 2.3. Statistical Analysis

**Figure 1:**
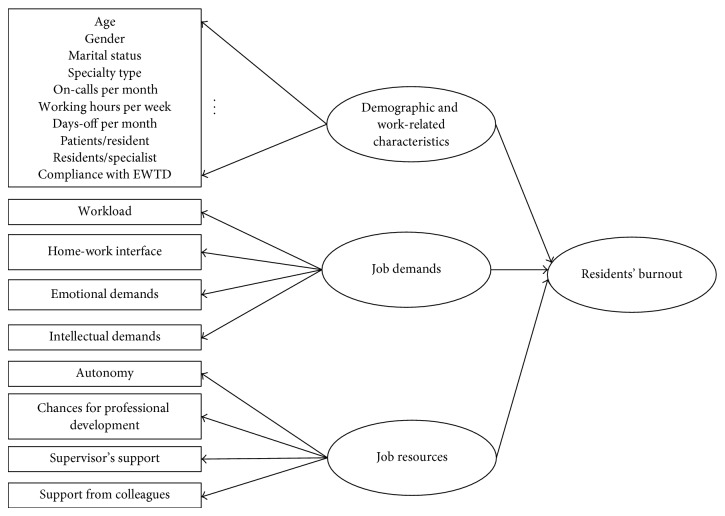
Conceptual model of residents' burnout adopted in our study.

The conceptual diagram of the model adopted in our study is shown in Figure 1. A database was developed using the statistical software package SPSS (version 16.0 for Mac; SPSS). Frequencies and descriptive statistics were examined for each variable. Statistical comparisons were performed between the “burned out” residents and the “nonburned out” residents concerning demographic characteristics, job demands and resources, and other work-related factors. Dichotomous variables were compared with the chi-square test, normally distributed continuous variables by using Student's *t*-test and nonnormally distributed continuous variables by using Mann-Whitney's *U* test.

Where statistically significant differences were found, correlations were computed to identify variables that could be entered into a logistic regression model to identify factors associated with outcome and estimate the probability that a case is a member of one of the categories of the dependent variable. Where variables were correlated, the most clinically relevant was selected for inclusion in the model. These variables were entered as independent variables and presence of burnout was entered as the dependent variable. A value of *P* < 0.05 for the Wald criterion was considered to denote regression coefficients significantly different from zero.

In order to identify influential cases and outliers, Cook's distances (>1.0) and standardized residuals (>3.0 in absolute value) were, respectively, computed and examined. Multicollinearity (occurring when some independent variables are highly intercorrelated) and numerical problems in the solution were detected by examining the standard errors (>2.0) for the estimated coefficients (*B*). The results are shown as odds ratio (OR) with 95% confidence intervals (CIs) for ORs. The fit of the models was judged by the Hosmer-Lemeshow goodness-of-fit statistic. The models were considered acceptable if *P* > 0.05 for model chi-square, given that a better model fit was indicated by a smaller difference in the observed and predicted values of the dependent variable, and a nonsignificant chi-square value. Two-way classification tables were used in order to summarize the fit between the actual and predicted group membership, that is how many participants the regression model correctly classified as experiencing burnout or not and how many participants the regression model incorrectly classified as experiencing burnout or not. Based on the classification table when all independent variables had been entered in the equation, the classification accuracy rate was obtained based on the sum of the main diagonal numbers, divided by the total sample size.

In order to look in to each dimension of burnout separately comparisons of each burnout dimension's score for marital status, type of specialty, and EWTD compliance were tested by Student's *t*-test and one-way analysis of variance. Correlations between each burnout dimension's score, trainees/specialist ratio and job demands, and resources subscores were examined by Pearson's correlation. Factors associated with each burnout dimension's score were identified by a general linear model with adjustment for age and gender. Statistical significance was defined as *P* < 0.05.

## 3. Results

### 3.1. Response

Of the 373 residents employed, 83 did not meet inclusion criteria.  Table 1 summarizes the list of all different specialties included in the study with the corresponding eligible residents and their response rates. Of the 290 eligible residents, 263 (overall rate 90.7%) responded. The response rate of residents of medical specialties was 91.4%, of surgical specialties 87.8% and of laboratory specialties 93.3%.

### 3.2. Sociodemographic and Work-Related Characteristics

The study population had a mean age of 33.5 (SD 3.3) years, 141 (53.6%) were male and 170 (64.6%) were single. The mean working hours per day, not including on-call duties, were 8.0 (SD 1.5) hours, the mean number of days-off was 1.6 (SD 1.7) per month, the mean number of on-calls was 6.9 (SD 1.6) per month, and the mean working hours per week, including on-call duties, was 60.9 (SD 14.7) per week. The number of doctors exceeding the limit set up by the European working time directive was calculated to be 142 (61.2%).

The mean remaining time to complete training was 26.0 (SD 15.2) months, the mean time between medical graduation and beginning of specialty training was 3.0 (SD 1.8) years, the mean residents/specialist ratio was 1.9 (SD 1.0), and the mean patients/resident ratio was 3.0 (SD 2.0). The latter referred only to specialties with immediate contact with patients.

### 3.3. Internal Consistency Reliability of the Scales

We tested the internal consistency reliability for both questionnaires regarding the eight job demands and resources scales. For the study population, Cronbach's alpha was 0.79 for support from colleagues, 0.89 for supervisor's support, 0.81 for workload, 0.74 for intellectual demands, 0.82 for emotional demands, 0.77 for home-work demands' interface, 0.74 for autonomy, and 0.78 for opportunities for professional development. Regarding the Maslach Burnout Inventory, Cronbach's alpha was 0.88 for EE, 0.71 for DP, and 0.77 for PA.

### 3.4. Total Burnout-Univariate Analysis

Table 2 summarizes the cutoff points according to the 25th and 75th percentile that formed groups of low, moderate, and high levels of burnout for each MBI dimension. The group of residents with moderate levels of burnout was represented by half of the participants as it included residents whose scores lay between the 25th and the 75th percentile. Out of the 263 residents who participated in the study, 134 (51.0%) presented high levels of burnout in at least one dimension, 48 (18.3%) in at least two dimensions, and 13 (4.9%) in all three dimensions. As mentioned above, residents who had high scores on the EE and the DP dimensions, or residents who scored high on the EE and low on the PA dimensions, were considered to be “burned out.” In total 38 (14.4%) residents were found to be burned out.

Table 3 shows the characteristics of those with burnout syndrome and those without it. The “burned out” residents worked more frequently in surgical specialties, worked more hours per week, and complied less with the European working time directive. The residents/specialist ratio was lower among the “burned out” residents, while the patients/resident ratio was significantly higher. Regarding the JD-R characteristics, “burned out” residents had less support from their supervisor, experienced increased workload, reported more intellectual and emotional demands, and had experienced conflicts in the interface between familial and professional life, less autonomy at work, and less chances of professional development.

### 3.5. Total Burnout-Multivariate Analysis

The following independent variables were entered into the multivariate logistic regression model: gender, age, compliance with the EWTD, residents/specialist ratio, type of specialty, supervisor's support, workload, emotional demands, intellectual demands, home-work demands interface, autonomy, and opportunities for professional development. Working hours and days off were not entered as they were highly correlating with compliance with the EWTD. Patients/residents ratio was not entered, as it was not applicable for laboratory specialties. There was one case with large standardized residual (equal to −5.59) and Cook's distance (equal to 1.83) that was considered an outlier and was excluded from this analysis. Multicollinearity or numerical problems were not detected, as none of the independent variables had a large standard error. The model chi-square value indicated that there was a statistically significant overall relationship between the dependent variable and the set of independent variables (*χ* ^2^ = 66.311, *df* = 13, *P* < 0.001). The chi-square value associated with the Hosmer-Lemeshow test (*χ* ^2^ = 3.774, *df* = 8, *P* = 0.877) indicated a good overall model fit. The Negelkerke *R* ^2^ was equal to 0.459.

Table 4 shows that, according to the Wald criterion, the unstandardized coefficients for the residents/specialist ratio (*B* = −0.891), for “home-work interface” (*B* = 0.227), for “autonomy” (*B* = −0.463), and for “opportunities for professional development” (*B* = −0.500) appeared to be significantly different from zero, using a significance level of 0.05. Each increased point in the JD-R questionnaire score regarding home-work demands' interface was associated with an increase in the odds of burnout by 25.5%. Conversely, each increased point for autonomy, opportunities in professional development, and each extra resident per specialist were associated with a decrease in the odds of burnout by 37.1%, 39.4%, and 59.0%, respectively. The classification accuracy rate was equal to 88.6%, supporting the utility of the model.

### 3.6. Burnout Dimensions-Univariate Analysis

The univariate analysis of EE score in relation to demographics, work-related characteristics, and JD-R variables showed that single residents had significantly higher EE scores. The following JD-R variables showed negative correlation with EE score; social support, supervisor's support, autonomy, opportunities for professional development when the following JD-R variables showed positive correlation with EE score; workload, intellectual demands, emotional demands, and home-work demands' interface.

The univariate analysis of DP score in relation to demographics, work-related characteristics, and JD-R variables showed that residents violating the EWTD, working in surgical specialties, and being single had significantly higher DP scores. The following JD-R variables showed negative correlation with DP score; intellectual demands, autonomy, and opportunities for professional development when the home-work demands' interface showed positive correlation with DP score.

The univariate analysis of PA score in relation to demographics, work-related characteristics, and JD-R variables showed that residents not violating the EWTD and working in laboratory specialties had significantly lower PA scores. The following JD-R variables showed positive correlation with PA score; social support, supervisor's support, intellectual demands, and opportunities for professional development, when the home-work demands' interface showed negative correlation with DP score.

### 3.7. Burnout Dimensions-Multivariate Analysis

A general linear model analysis of the factors associated with burnout dimension scores is shown in Table 5. After controlling for age and gender, variables that predicted a high level of EE included marital status, supervisor's support, home-work demands' interface, autonomy, and opportunities for professional development. After controlling for age and gender, variables that predicted a high level of DP included marital status, intellectual demands, home-work demands' interface, and autonomy. Finally, after controlling for age and gender, variables that predicted a high level of PA included EWTD compliance, intellectual demands, and opportunities for professional development.

## 4. Discussion

The results of this cross-sectional study showed that burnout is not uncommon among residents, in the largest hospital of Greece. More than half of the residents who participated in the study presented with high levels of burnout in at least one burnout dimension. When a more strict definition of burnout was applied we found that almost 1 out of 7 resident suffered from burnout syndrome.

Other similar studies have shown that percentage of burnout among doctors in training ranges from 18 to 82% [18]. However, the instruments used to measure burnout and the exact definition of burnout syndrome varied from study to study. Most studies have used MBI: the research edition [23–25], the second version [26–28], or the third version [4, 5, 29–33]. However, other tools such as the Hacker and Reinhold Stresses and Strains Screening in Human Services [34], the 30-item Staff Burnout Scale for Health Professionals [35], and the Utrecht Burnout Scale [36] have been used to measure burnout. Brenninkmeijer and VanYperen [22] have suggested that the “EE + 1” criterion results in a fairly small chance of an inaccurate qualification of burnout (i.e., a false positive). We also used this criterion, as this is the most effective way of diagnosing burnout. However, if we had used the stricter definition that a resident suffers from burnout syndrome if he/she presents with high level of burnout in all MBI dimensions, then the burnout rate in our population would be 4.9%. This is the lowest percentage of burnout among residents reported as yet.

It should be mentioned, however, that burnout is not a question of “all or nothing.” It is a multidimensional construct, which has many stages [37]. Using cutoffs for dichotomizing variables more or less is employed only as it helps to quantify the impact of independent variables and makes results more understandable. In our study, apart from treating burnout as a dichotomous variable we also proceeded to further multivariate analyses of each dimension as a continuous variable.

To our knowledge this is one of the few studies that have used the JD-R model to identify factors associated with burnout among doctors in training. The JD-R model has been used in healthcare working environments in studies among dentists [38], nurses [39–42], and palliative care volunteers [43]. In these studies, it has been shown that demands are more predictive of burnout than resources [39]. In particular time pressure [42] and subsequent increased workload show a significant relationship with burnout. Our univariate analysis showed that, among residents, both resources at work (supervisor's support, autonomy, and opportunities for professional development) and job demands (intellectual demands, emotional demands, workload, and home-work demands interface) are related to burnout. The multivariate analysis showed that opportunities for professional development, autonomy, and home-work demands interface were significantly related to burnout. Interestingly, among the work-related factors only the lower resident per specialist ratio was significantly related to burnout among Greek medical residents. The Greek healthcare system relies heavily on the service of medical residents who remain in the hospital overnight, while the consultant/specialist is often on call from home. Greek residents report that their daily residency schedule is hampered by too much paperwork and menial tasks, which is related to increased EE [44]. Thus, a lower resident per specialist ratio may lead to workload and burnout for the few residents available to provide both hospital care and “scutwork” (tasks that are tedious and monotonous or trivial and menial).

One other advantage of our study is that our study population included only residents. It is known that only a very small percentage of burnout studies focuses on burnout among medical residents, despite the fact that young doctors in training work in demanding environments characterized by heavy patient loads and long, irregular working environments, and are highly dependent on supervisor's evaluations to complete their training [22]. Most of the studies that have investigated burnout among residents have been carried out only in one specialty. The limited number of studies that have been conducted among residents of different specialties have not only covered a limited number of specialties such as eight [31] to thirteen [10] different specialties but have had poor response rates such as 35% [10] to 38% [31]. We managed to cover twenty-five different specialties and achieve a nearly 91% response rate.

Only one out of three residents of our study group complied with the EWTD, a fact that did seem to affect burnout levels. A Greek study designed to assess the prevalence of burnout of medical residents in Greece, prior to implementation of the EWTD, had estimated that 31.8% of the residents showed high levels of burnout in all three dimensions [44]. Despite the fact that the methodology used in that study was completely different from ours, given that it included residents from eight different hospitals in three different cities, that percentage of burned out residents was six times higher than in our study. This may indicate that the implementation of EWTD can lead to reduced burnout levels and that if we manage to comply better with the EWTD then the burnout levels may continue to fall. This hypothesis is concordant with the findings of a study performed among pediatric residents, where it was shown that after the implementation of work hour limits for resident physicians introduced by the Accreditation Council for Graduate Medical Education fewer residents were burned out [45].

There are a few limitations that need to be mentioned. Firstly, in this study we used our own sample as normative population and therefore we used cutoff points based on this sample, as normative values for the Greek population are not yet available. Secondly, the study was cross-sectional, meaning that no firm conclusions regarding the causality of relationships can be drawn. Second, the number of patients in each specialty was relatively small, which did not allow us to study the relationship between specialty and burnout but only examine, through the logistic regression analysis, whether the specialty type (i.e., medical, surgical, or other) was related to burnout. Moreover, despite the fact that we covered 25 different specialties, we could not gather data for obstetrics, gynecology, and pediatric specialties, as this hospital did not have such departments. Finally, our study group comprised residents of one hospital, and results might not be generalisable to other settings.

Preventing burnout syndrome is important not only for the health service employees who suffer but also for the health service users who can become victims of it. It has been reported that residents regularly self-report making errors throughout their residency [46]. Once present, burnout tends to persist through residency [3], while it can be contagious and may cross over from one health professional to another [47]. Therefore investing in early interventions is the key to minimizing or preventing burnout. Given that medical student burnout is frequent, the interventions should start being implemented in the medical schools, as burnout can even be present prior to the beginning of specialty training and even affect the specialty choice [48, 49]. In our study, we found that one of the important correlates of burnout was home-work demands' interface. Given that low resources and high demands in one domain are likely to worsen outcomes in the other domain (work-home conflict) [50, 51], interventions should target residents with burnout to help them optimize social support networks and health organizations should adopt family-friendly policies in order to reduce burnout. Another important factor found was “opportunities for professional development.” Continuing medical education may reduce job distress [52] and therefore advanced training courses and better chances of being involved in research projects during the specialty training may reduce residents' burnout levels and enhance the quality of care they provide.

Finally, inadequate support from the supervisor has been identified as a factor associated to burnout, and especially EE. As medical residents are in training, it is expected that they might be limited by their lack of experience. A good support team would not only help to reduce stress, but also to improve quality of care.

## Figures and Tables

**Table 1:** List of specialties included in the study with the corresponding eligible residents and their response rates, grouped by category.

Type	Employed residents	Eligible residents	Responded residents	Response percentage
Medical specialties				
Cardiology	26	19	18	95%
Dermatology	7	7	7	100%
Endocrinology	9	8	8	100%
Gastroenterology	10	8	7	88%
Hematology	13	10	9	90%
Internal medicine	42	32	28	88%
Nephrology	10	8	6	75%
Neurology	19	19	19	100%
Medical oncology	4	4	3	75%
Psychiatry	13	12	12	100%
Respiratory medicine	14	8	6	75%
Rheumatology	7	5	5	100%
Surgical specialties				
Anesthesiology	21	10	7	70%
Cardiothoracic surgery	14	14	14	100%
Ear-nose-throat	6	6	5	83%
General surgery	33	25	22	88%
Neurosurgery	8	7	7	100%
Ophthalmology	6	6	5	83%
Plastic surgery	4	4	3	75%
Trauma and orthopedics	15	13	12	92%
Urology	8	5	4	80%
Other specialties				
Microbiology	36	20	17	85%
Nuclear medicine	5	4	4	100%
Pathology	20	18	17	94%
Radiology	23	18	18	100%
Total	**373**	**290**	**263**	**91%**

**Table 2:** Cutoff points and categories of low, moderate, and high levels of burnout per burnout dimension.

	Low	Moderate	High
MBI dimensions			
Emotional exhaustion	≤16	17–33	≥34
Sense of personal accomplishment	≥39	30–38	≤29
Depersonalization (cynicism)	≤4	5–12	≥13

**Table 3:** Characteristics of “burned out” and “nonburned out” residents.

	Burnedout residents (*n* = 38)	Nonburned out residents (*n* = 225)	*P*
Demographics			
Age in years	32.8 (2.3)	33.6 (3.4)	0.162
Male gender (%)	24 (63.2)	117 (52.0)	0.202
Marital status			0.170
Single (%)	29 (76.3)	140 (62.2)	
Married (%)	9 (22.0)	76 (34.2)	
Divorced (%)	0 (0.0)	9 (4.1)	
Work-related characteristics			
Type of specialty			**0.015** ^*^
Medical (%)	13 (34.2)	116 (51.5)	
Surgical (%)	19 (50.0)	60 (26.7)	
Other (%)	6 (15.8)	49 (21.8)	
Months remaining to complete training	26.6 (15.0)	25.9 (15.3)	0.789
Years between graduation and beginning of training	2.9 (1.9)	3.0 (1.8)	0.730
Working hours per day (not including on-call duties)	8.4 (1.8)	7.9 (1.5)	0.061
Working hours per week (including on-call duties)	66.7 (13.1)	60.0 (14.7)	**0.016** ^*^
EWTD complied (%)	8 (21.1)	93 (41.3)	**0.034** ^*^
On-calls per month	7.0 (1.7)	6.9 (1.6)	0.910
Days-off per month	0.8 (1.3)	1.8 (1.7)	**0.002** ^**^
Residents/specialist ratio	1.5 (0.6)	2.0 (1.0)	**0.007** ^**^
Patients/resident ratio	3.2 (2.3)	2.2 (2.1)	**0.005** ^**^
Job demands and resources			
Support from colleagues	11.1 (3.0)	11.6 (2.5)	0.247
Supervisor's support	11.2 (4.8)	14.2 (5.0)	**0.001** ^**^
Workload	17.3 (2.5)	16.0 (2.9)	**0.011** ^*^
Intellectual demands	13.5 (1.9)	12.8 (1.9)	**0.042** ^*^
Emotional demands	21.3 (4.1)	19.4 (4.5)	**0.017** ^*^
Work-home demands conflict	10.1 (2.5)	8.0 (2.6)	**<0.001** ^***^
Autonomy	6.6 (2.0)	8.1 (2.0)	**<0.001** ^***^
Opportunities for professional development	7.1 (1.9)	9.1 (1.9)	**<0.001** ^***^

Noncontinuous variables are given as percentages. Continuous variables are presented as mean values with their corresponding standard deviation. EWTD, European working time directive. ^*^ *P* < 0.05; ^**^ *P* < 0.01; ^***^ *P* < 0.001.

**Table 4:** Logistic regression of residents' burnout on demographic and work-related variables, as well as job demands and job resources.

Variable	*B*	SE	Wald	*P*	OR (95% CI)
Male gender	0.364	0.542	0.451	0.502	1.439 (0.497–4.163)
Age (per year)	−0.063	0.086	0.527	0.468	0.939 (0.793–1.113)
Type of specialty			2.159	0.340	
Surgical	0.224	0.628	0.127	0.722	1.251 (0.365–4.285)
Lab/other	−1.084	0.926	1.372	0.241	0.338 (0.055–2.075)
Noncompliance with the EWTD	−0.288	0.663	0.189	0.664	1.334 (0.364–4.889)
Residents/specialist ratio (per extra resident)	−0.891	0.403	4.881	**0.027** ^*^	0.410 (0.186–0.904)
Supervisor's support	−0.068	0.057	1.423	0.233	0.934 (0.836–1.045)
Workload	−0.009	0.123	0.006	0.939	0.991 (0.779–1.260)
Intellectual demands	0.294	0.193	2.310	0.129	1.342 (0.918–1.960)
Emotional demands	0.072	0.064	1.262	0.261	1.075 (0.948–1.218)
Work-home demands interface	0.227	0.104	4.771	**0.029** ^*^	1.255 (1.024–1.539)
Autonomy	−0.463	0.154	9.092	** 0.003** ^**^	0.629 (0.466–0.850)
Opportunities for professional development	−0.500	0.153	10.754	**0.001** ^**^	0.606 (0.450–0.818)

CI, confidence interval ^*^ *P* < 0.05; ^**^ *P* < 0.01.

**Table 5:** General linear model analysis of the factors associated with burnout dimensions' scores.

Variable	EE		DP		PA	
	*B*	Beta	*B*	Beta	*B*	Beta
Sex	−0.904	−0.41	−2.938	−0.260^***^	−0.597	−0.042
Age	−0.126	−0.38	−0.122	−0.067	0.272	0.129^*^
Marital status	−2.191	−0.109^*^	−1.513	−0.148^*^		
EWTD compliance			0.696	0.060	2.157	0.148^*^
Specialty type			0.546	0.076	−1.231	−0.136^*^
Social support	−0.119	−0.028			−0.047	−0.017
Supervisor support	−0.286	−0.131^*^			0.136	0.097
Workload	0.374	0.097				
Intellectual demands	0.115	0.020	−0.373	−0.125^*^	0.691	0.183^**^
Emotional demands	0.067	0.027				
Work-home demands conflict	1.621	0.395^***^	0.516	0.248^***^	−0.317	−0.121
Autonomy	−0.676	−0.125^*^	−0.501	−0.173^**^		
Opportunities for professional development	−1.191	−0.220^***^	−0.325	−0.117	0.840	0.240^***^

EE, emotional exhaustion; DP, depersonalization; PA, sense of personal accomplishment. ^*^ *P* < 0.05; ^**^ *P* < 0.01; ^***^ *P* < 0.001.
